# Do Biobank Recall Studies Matter? Long-Term Follow-Up of Research Participants With Familial Hypercholesterolemia

**DOI:** 10.3389/fgene.2022.936131

**Published:** 2022-07-19

**Authors:** Miriam Nurm, Anu Reigo, Margit Nõukas, Liis Leitsalu, Tiit Nikopensius, Marili Palover, Tarmo Annilo, Maris Alver, Aet Saar, Toomas Marandi, Tiia Ainla, Andres Metspalu, Tõnu Esko, Neeme Tõnisson

**Affiliations:** ^1^ Institute of Genomics, University of Tartu, Tartu, Estonia; ^2^ Institute of Technology, University of Tartu, Tartu, Estonia; ^3^ Department of Biotechnology, Institute of Molecular and Cell Biology, University of Tartu, Tartu, Estonia; ^4^ Department of Genetic Medicine and Development, University of Geneva, Geneva, Switzerland; ^5^ Department of Cardiology, Institute of Clinical Medicine, University of Tartu, Tartu, Estonia; ^6^ Cardiology Centre, North Estonia Medical Centre, Tallinn, Estonia; ^7^ Genetics and Personalized Medicine Clinic, Tartu University Hospital, Tartu, Estonia

**Keywords:** familial hypercholesterolemia (FH), lipid lowering treatment, recall-by-genotype, participant survey, biobank to practice, long-term survey, pharmacogenomic risk assessment

## Abstract

Recall-by-genotype (RbG) studies conducted with population-based biobank data remain urgently needed, and follow-up RbG studies, which add substance to this research approach, remain solitary. In such studies, potentially disease-related genotypes are identified and individuals with those genotypes are recalled for consultation to gather more detailed clinical phenotypic information and explain to them the meaning of their genetic findings. Familial hypercholesterolemia (FH) is among the most common autosomal-dominant single-gene disorders, with a global prevalence of 1 in 500 (Nordestgaard et al., Eur. Heart J., 2013, 34 (45), 3478–3490). Untreated FH leads to lifelong elevated LDL cholesterol levels, which can cause ischemic heart disease, with potentially fatal consequences at a relatively early age. In most cases, the pathogenesis of FH is based on a defect in one of three LDL receptor-related genes–*APOB*, *LDLR*, and *PCSK9*. We present our first long-term follow-up RbG study of FH, conducted within the Estonian Biobank (34 recalled participants from a pilot RbG study and 291 controls harboring the same *APOB*, *LDLR*, and *PCSK9* variants that were included in the pilot study). The participants’ electronic health record data (FH-related diagnoses, lipid-lowering treatment prescriptions) and pharmacogenomic risk of developing statin-induced myopathy were assessed. A survey was administered to recalled participants to discern the impact of the knowledge of their genetic findings on their lives 4–6 years later. Significant differences in FH diagnoses and lipid-lowering treatment prescriptions were found between the recalled participants and controls (34 and 291 participants respectively). Our study highlights the need for more consistent lipid-lowering treatment adherence checkups and encourage more follow-up RbG studies to be performed.

## Introduction

Recall-by-genotype (RbG) studies have proven value as population-based biobank studies conducted with a “genotype-first” approach. Individuals harboring high-risk genetic variants are first identified by genomic data and then stratified based on clinical phenotypic information and electronic health record (EHR) data, with the identification of groups in need of medical attention. Such studies have been performed successfully at the Estonian Biobank (EstBB) (Leitsalu et al., 2016; Alver et al., 2018; Leitsalu et al., 2020) and elsewhere (Haukkala et al., 2013; Stessman et al., 2014; Ormondroyd et al., 2020), and have been shown to benefit those carrying deleterious genetic variants. Although RbG studies are gaining more traction in the scientific community (Corbin et al., 2018), long-term follow-up studies of this type remain scarce due to the novelty of the concept.

The RbG approach is most beneficial when applied to the investigation of disorders that are present in sufficient frequency in the study population and for which actionable treatment options are available. Familial hypercholesterolemia (FH) is among the most common known autosomal-dominant single-gene disorders, with a prevalence of 1 in 500 globally and 1 in 200 in Northern European populations (Nordestgaard et al., 2013; Benn et al., 2016). In most cases, the pathogenesis of FH is caused by a defect in one of the three low-density lipoprotein receptor-related genes: *LDLR*, *APOB*, and *PCSK9* (Berberich and Hegele, 2019).

Despite its high rate of occurrence worldwide and readily available treatment (Gidding et al., 2015), FH is often underdiagnosed and undertreated; many patients with this disease receive suboptimal or delayed treatment, without the necessary attention given to genetic factors that may influence its course. Thus, treatment goals remain inadequate for long periods and, without genetic diagnosis, may not follow established guidelines (Cuchel et al., 2014). When left untreated, FH leads to the lifelong elevation of low-density lipoprotein cholesterol (LDL-C) levels, which can cause ischemic heart disease, potentially resulting in early death (Nordestgaard et al., 2013; Gidding et al., 2015). Moreover, lipid-lowering treatment (LLT) adherence tends to be inadequate among FH patients, despite its proven efficacy (Casula et al., 2016; Langslet et al., 2021). This reflects a further need for continuous FH education and follow-through.

Provided that the genotype data is available, primary prevention of FH could also start from pre-symptomatic genetics-first screening applied on a general population-wide biobank, and then be directed to families via cascade screening. To increase effectiveness, universal blood lipid measurements at a pre-defined (and still reasonably early) age should complement the strategy for those not yet reached via family members (Groselj et al., 2022).

The objectives of this follow-up study were to assess the long-term impacts of an RbG FH study (Alver et al., 2018) by surveying recalled participants 4–6 years after the initial return of their results at the EstBB, and to analyze FH-related diagnostic and treatment adherence data retrieved from the healthcare system from a period of up to 18 years encompassing the study period. The findings provide a longer-term perspective on the RbG study approach and the Estonian healthcare systems’ handling of such information.

## Materials and Methods

The original study from which this follow-up study derived was conducted in 2016–2018 with the objectives of identifying EstBB participants with FH-associated gene variants based on sequencing data, and recalling carriers for biochemical analyses and medical examination to detect early FH manifestations (Alver et al., 2018). Additionally, family members were recruited through cascade screening, resulting in a final cohort of 41 confirmed FH variant carriers. All participants received feedback at the end of their visits, with explanation of their genetic findings and final diagnoses and recommendations for further treatment plans.

### Cohort Overview

The EstBB currently has more than 200,000 participants (about 20% of Estonia’s adult population) for whom multilevel molecular and phenotype data have been collected. These data are not only used by the scientific community, but also have potential for practical population-level healthcare applications. When joining the biobank, all participants provided broad written consent, allowing the EstBB to re-contact them and update their data through EHR and national health registry linkage (Leitsalu et al., 2020).

**FIGURE 1:**
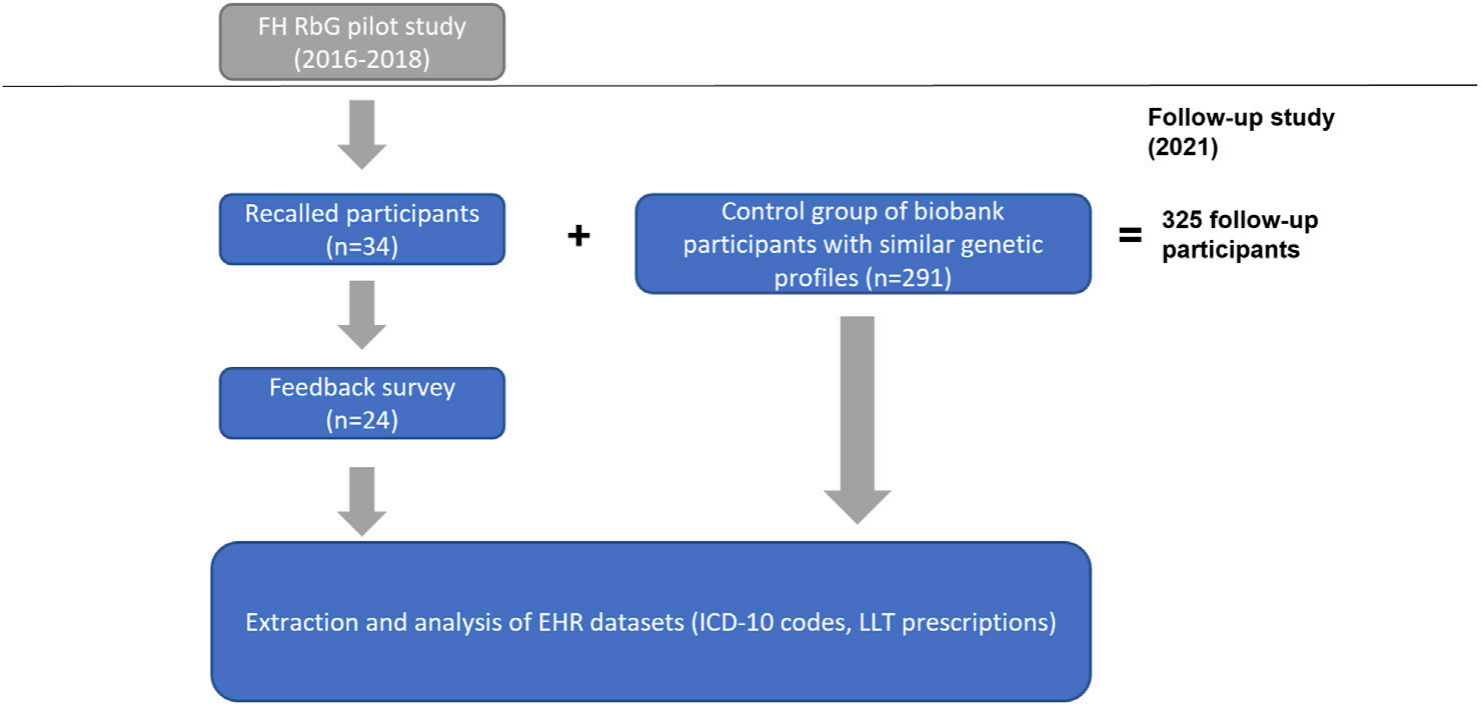
The workflow of the study. The initial cohort of recalled participants originated from the FH RbG pilot study conducted by Alver et al. in 2016–2018. Recalled participants who had also signed up as biobank participants by Fall 2021 were eligible to participate in the follow-up.

In the pilot RbG study (Alver et al., 2018), high-coverage sequencing data (available for 4,776 individuals) and Sanger sequencing were used to identify and confirm FH-associated variant carriers (*n* = 51). Thirty-four of these recalled individuals who continued to be EstBB participants were included in this follow-up study. Using genotype and imputed data (available for 201,146 EstBB participants), we identified carriers with similar genetic backgrounds (based on 14 genetic variants considered in the pilot study; Alver et al., 2018) to form a control group (*n* = 291) of individuals not yet recalled for the disclosure of their carrier status (Supplementary Figure S1). Clinical histories from linked EHRs were available for individuals in both groups; the workflow of the study can be seen in Figure 1.

### Genotyping and Imputation

Genome and exome sequencing data for the primary study cohort (*n* = 4,776), including custom quality-control and variant-annotation pipeline outputs (Alver et al., 2018), were retrieved. Genotyping of the EstBB samples was performed using Illumina global screening arrays (v. 1.0 and 2.0; Illumina Inc., San Diego, CA, United States) at the Core Genotyping Lab of the Institute of Genomics, University of Tartu, Estonia. Quality control criteria for the exclusion of individual data from genotype-based analyses were call rate < 95% and sex mismatch between genotype and phenotype data. Before imputation, the variants were filtered by call rate <95%, Hardy-Weinberg equilibrium *p* < 1 × 10^–4^ (for autosomal variants), and minor allele frequency <1%. The Eagle software (v. 2.3; Loh et al., 2016) was applied for prephasing and the Beagle software (v. 28Sep18.793; Browning and Browning, 2007) was used for imputation based on the Estonian population-specific reference panel built from 2,297 genome sequencing samples (Mitt et al., 2017). We screened the available direct SNP genotyping and imputed data for 11 FH-associated variants considered by Alver et al. (2018). Priority was given to datasets in which individuals’ carrier status was identified from both directly genotyped and imputed data (*n* = 225). A subset of individuals in whom the variants of interest were identified either from directly genotyped or imputed data (*n* = 66) were added for validation. Sanger sequencing confirmed FH-associated variants in a total of 291 persons.

### EHR Data Linkage

EHRs covering a period of up to 18 years (2004–2022) were retrieved for all study participants as described by Leitsalu et al. (2015). All biobank participants had consented to make their EHRs available for scientific research. The participants’ general information, ICD-10 diagnosis codes, and drug prescription data (ATC/DDD codes, dosage, and purchase) were obtained from the EHRs and used for analysis.

### Participant Survey

Of the 34 participants recalled in the pilot RbG study, 32 were recontacted by mail and asked to fill out a questionnaire (Supplementary Data 1) 4–6 years after their initial recall. The remaining two recalled participants were excluded from the survey because of outdated contact information and death in 2018, respectively. The participants were given 14 days to return the questionnaire; in the event of nonresponse, they were contacted by telephone. Further nonresponse was taken to indicate that participants had declined to take part in the follow-up study.

The questionnaire consisted of 40 items regarding the perceived usefulness of the genetic feedback that participants had received (including their understanding of the information), the impact that knowledge of their genetic findings had had on their lives (including adherence to their treatment plans), the participants’ assessment of the healthcare system’s efficiency in integrating this information, and whether they had experienced potential FH complications after their feedback visits. Item responses were converted to a numeric scale ranging from 1–5, with values <3 signifying negative attitudes and those >3 signifying positive attitudes toward the item statements. The survey was developed in Estonian and contained questions inspired by published survey instruments (Marteau and Bekker, 1992) and analogous studies (Brehaut et al., 2003).

### LLT Adherence

To visualize LLT adherence, a Gantt chart was created in Python using the plotly^1^ library (v. 5.6.0, 2015; Plotly Technologies Inc., Montreal, QC, Canada) displaying the periods of LLT use from 2004 to March 2022 among recalled participants with LLT prescriptions. These periods were defined from the date of drug purchase, recorded in the drug prescription registry, to the end date of prescription consumption, calculated as the number of packages multiplied by the number of pills and added to the purchase date as the number of days (thus assuming that participants consumed one tablet per day). This formula was adjusted as needed on a case-by-case basis; for example, when a participant purchased several prescriptions on the same day, the start dates for subsequent packages were adjusted based on the consumption end dates for previous packages. Consistent LLT use was defined as use without a gap >6 months.

### Pharmacogenomics

EstBB participants’ genotype data were translated into pharmacogenomic risk phenotypes for 11 clinically important pharmacogenes using a pipeline developed by Reisberg et al. (2019). In the current study, results for the *SLCO1B1* gene were used to assess the risk of statin-induced side effects.

Pharmacogenomic profiles were created for the 27 recalled LLT users and included the following information: age, sex, FH-associated genetic variant, LLT adherence, previous FH-related medical history (ICD-10 codes), statin-adjusted (divided by 0.8 and 0.7, respectively) total cholesterol and LDL-C values measured during primary feedback visits, statin-induced myopathy risk, and an overview of survey responses about treatment plan adherence and health (Supplementary Table S1). LLT adherence was represented graphically using Python with the matplotlib library (v. 3.3.2; Hunter, 2007). Differences between the recalled and control groups were examined with Langsrud^2^’s two-tailed Fisher’s exact test calculator, which uses Agresti (1992) as a reference.

The protocol (and further amendments) for this study was approved by the Ethics Review Committee on Human Research of the University of Tartu and Estonian Committee on Bioethics and Human Research (approval no 1.1-12/3015).

## Results

### Cohort Overview

In total, this study included 325 EstBB participants harboring FH-associated variants [34 recalled participants from the original RbG study (Alver et al., 2018) and 291 non-recalled controls]. A summary of the FH-associated variants identified is provided in Table 1. Of the participants, 60.9% (*n* = 198) were carriers of an *APOB* variant, 42.3% (*n* = 123) harbored an *LDLR* variant, and 1.7% (*n* = 5) were carriers of a *PCSK9* variant. The FH-associated variants identified most frequently in the follow-up cohort were p.Arg3527Gln (rs5742904) in the *APOB* gene and p.Cys329Tyr (rs761954844) in the *LDLR* gene. Three rare FH-associated variants not described by Alver et al. (2018) were identified from sequencing data in this follow-up cohort. The general characteristics of the recalled and non-recalled groups were comparable (Table 2).

**TABLE 1:** FH-associated genetic variants detected in the follow-up cohort. Participants with *LDLR* p.Val436Ala (rs779732323) and *PCSK9* p.Ala103Ser (novel) were assigned to the control group because they declined visitation offered by Alver et al. (2018) in 2016–2018. *One individual had both *APOB* p.Arg3527Gln (rs5742904) and *LDLR* p.His250Arg (rs1256668310) variants. SNV, single nucleotide variant; rs, dbSNP reference number; RbG, recall by genotype; GS, genome sequencing; ES, exome sequencing.

Gene RefSeq Protein ID	SNP	RbG participants	Controls	Sum	Alver et al. (2018) (GS/ES)
APOB* ***NP_000375***		**22**	**176**	**198**	
	p.Arg3527Gln (rs5742904)	20	176	196	11
	p.Gly861Glu (rs1663664782)	1	0	1	
	p.Cys4217Alafs3* (novel)	1	0	1	
LDLR* ***NP_000518***		**11**	**112**	**123**	
	p.His250Arg (rs1256668310)	1	22	23	2
	p.Leu401His (rs121908038)	1	2	3	2
	p.Ala431Ser (rs28942079)	1	13	14	1
	p.Arg633His (rs754536745)	1	0	1	1
	p.Cys329Tyr (rs761954844)	3	72	75	5
	p.Arg215Cys (rs764042910)	2	1	3	1
	p.Gly396Ala (rs766474188)	1	0	1	1
	p.Arg115Cys (rs774723292)	1	0	1	1
	p.Val436Ala (rs779732323)	0	2	2	1
**PCSK9** ***NP_777596***		**1**	**4**	**5**	
	p.Arg357Cys (rs148562777)	1	3	4	1
	p.Ala103Ser (novel)	0	1	1	

**TABLE 2:** Cohort characteristics. RbG, recall-by-genotype; BMI, body mass index.

	RbG participants (*n* = 34)	Controls (*n* = 291)
Gender–female (%)	18 (52.9%)	186 (63.9%)
Age (range)	Median 49.5 (29–84)	Median 49 (21–100)
BMI (range)	Mean 25.5 (18.2–44.3)	Mean 26.3 (17.2–50.8)
Smoking (%)		
Never	19 (55.9%)	138 (47.4%)
Former	7 (20.6%)	70 (24.1%)
Current	8 (23.5%)	67 (23.0%)
Unknown	0	16 (5.5%)

### Clinical Profiles of Recalled and Non-Recalled FH Variant Carriers

The most relevant FH-associated findings from participants’ pharmacogenomic profiles are summarized in Table 3. The recalled cohort had significantly more diagnoses of lipoprotein metabolism disorders (ICD-10 E78*) and pure hypercholesterolemia (ICD-10 E78.0; the same code used for FH in clinical practice) than did the non-recalled group (94.1% vs. 67.0%, *p* < 0.001 and 82.4% vs. 46.0%, *p* < 0.001, respectively). Furthermore, the recalled group contained significantly more LLT users than did the non-recalled group (79.4% vs. 53.3%, *p* < 0.005). Statin-induced myopathy risks were similar in the recalled and non-recalled groups (higher than normal, 29.4% and 28.5%, respectively; much higher than normal, 2.9% and 2.4%, respectively).

**TABLE 3:** Principal FH-related findings. The statin risk warning is applicable only for simvastatin, atorvastatin, and rosuvastatin. *ICD-10 codes were reduced to three characters, with each code counted only once for each individual.

	RbG participants *n* = 34	Controls *n* = 291	*p*-value (<0.005)
All diagnoses* (excluding Z codes)	34 people with 1,413 diagnosis codes–on average 41.6 per person	291 people with 11,857 diagnosis codes–on average 40.7 per person	
Participants with E78 diagnosis code (including all subsets)	32 (94.1%)	195 (67.0%)	<0.001
Participants with E78.0 Pure hypercholesterolemia diagnosis code	28 (82.4%)	134 (46.0%)	<0.001
Users of any lipid lowering medication	27 (79.4%)	155 (53.3%)	<0.005
Statin side effect (myopathy) risk assessment according to genotype			
Normal risk	23 (67.6%)	201 (69.1%)	
Higher risk	10 (29.4%)	83 (28.5%)	
Much higher risk	1 (2.9%)	7 (2.4%)	

### LLT Adherence

**FIGURE 2:**
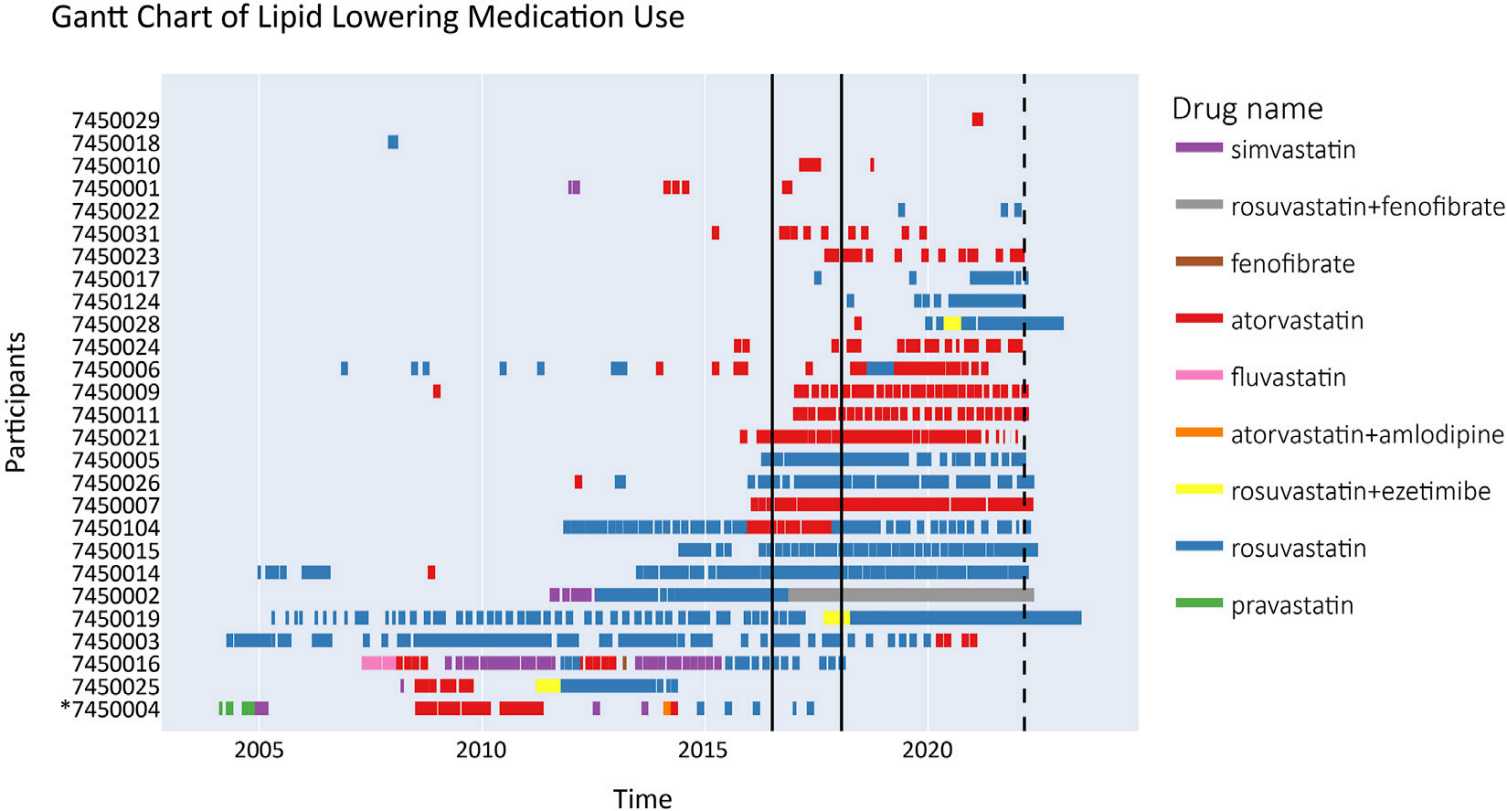
Gantt chart depicting lipid lowering treatment for recalled participants from 2004 until 2022 (*n* = 27). The time period between the two solid black lines signifies the period of RbG visits (2016–2018) in the Alver et al. study. The dashed line signifies the end of the drug prescription registry follow-up. The lines crossing the end of follow-up indicate purchase of a significant stock of medication in advance. Drug name here indicates the active substance of the prescribed LLT, not the brand name. Participant #7450004 passed away in 2018 (denoted by *).

The majority (59.3%) of participants continued LLT with the medications first prescribed to them. Atorvastatin was prescribed at least once to 70.4%, rosuvastatin to 63.0%, and medication combinations (rosuvastatin + fenofibrate or rosuvastatin + ezetimibe) to 14.8% of all participants (Figure 2).

The LLT users were allocated to three groups characterized by:

1) poor overall adherence (participants #7450029-#7450024 in the order listed in Figure 2; *n* = 11);
2) consistent LLT use at the time of the feedback visit or initiation shortly thereafter (participants #7450006-#7450019; *n* = 13); and
3) consistent LLT use for ≥2 years before the feedback visit, but termination for various reasons (participants #7450003-#7450004; *n* = 4).

More detailed data on LLT use are provided in Supplementary Table S1.

### Survey Results

The survey was returned by 24 (75%) recipients. Two participants contacted the EstBB to decline to participate in the survey, one because of a bad personal experience with statin-related side effects and another for unknown reasons.

**FIGURE 3:**
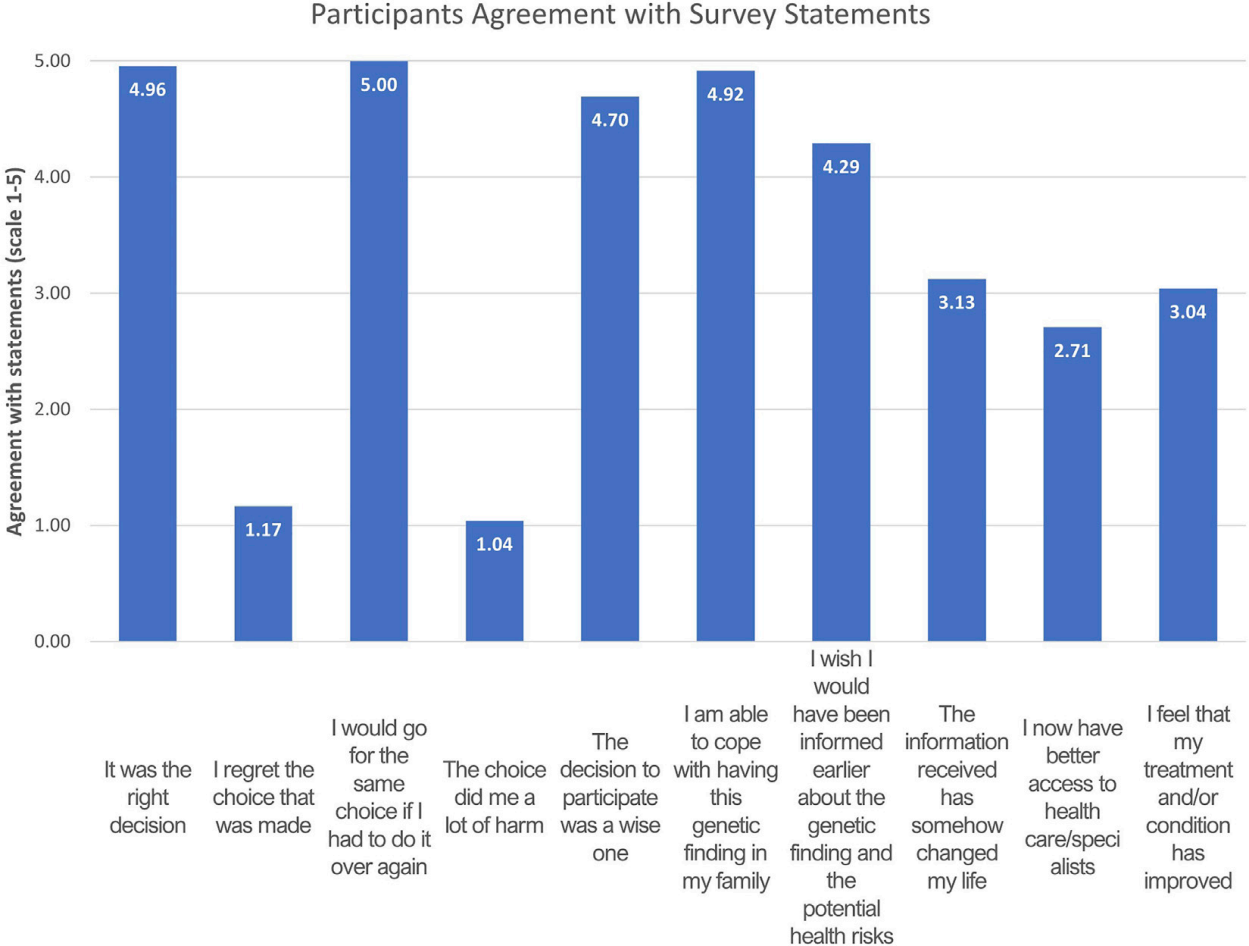
Mean values for answers given to the first portion of the RbG feedback survey where the participants were asked to rate their agreement with the following statements (*n* = 24). The answers were converted to numeric values according to the scale: “agree”-5, “slightly agree”-4, “unsure”-3, “slightly disagree”-2. “disagree”-1. Mean values above three signify agreement with the specific statement.

The respondents’ assessment of the feedback received was overwhelmingly positive. On average, they agreed that “it was the right decision” to attend the feedback visit (4.96/5), that they “wished to have been informed earlier about the genetic finding and the potential health risks” (4.29/5), and that they would “make the same choice if [they] had to do it over again” (5/5; Figure 3). The respondents largely agreed that the knowledge of their genetic finding did not cause them distress (“I am able to cope with having this genetic finding in my family,” 4.92/5). Average scores for the negative statements “I regret my choice” and “the choice did me a lot of harm” were 1.17/5 and 1.04/5, respectively. The respondents did not definitively agree or disagree with statements about access to healthcare and the improvement of their treatment and/or condition.

**FIGURE 4:**
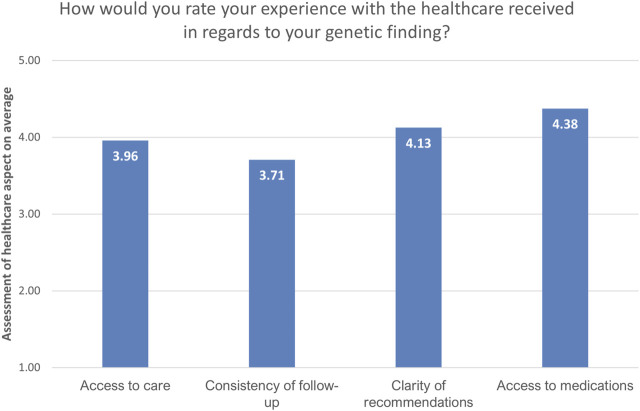
Overview of the assessment of the current state of the healthcare system by recalled participants (*n* = 24). The answers are presented as mean values of provided answers converted to numeric values: “very good”-5, “good”-4, “unsure”-3, “satisfactory”-2, “unsatisfactory”-1. Mean values above three signify satisfaction with the specific healthcare aspect.

Regarding aspects of the healthcare system in respect to their genetic findings, the respondents rated the consistency of follow-up the lowest (3.71/5), followed by access to healthcare (3.96/5). Access to medication was rated the highest (4.38/5), followed by the clarity of recommendations (4.13/5; Figure 4).

**FIGURE 5:**
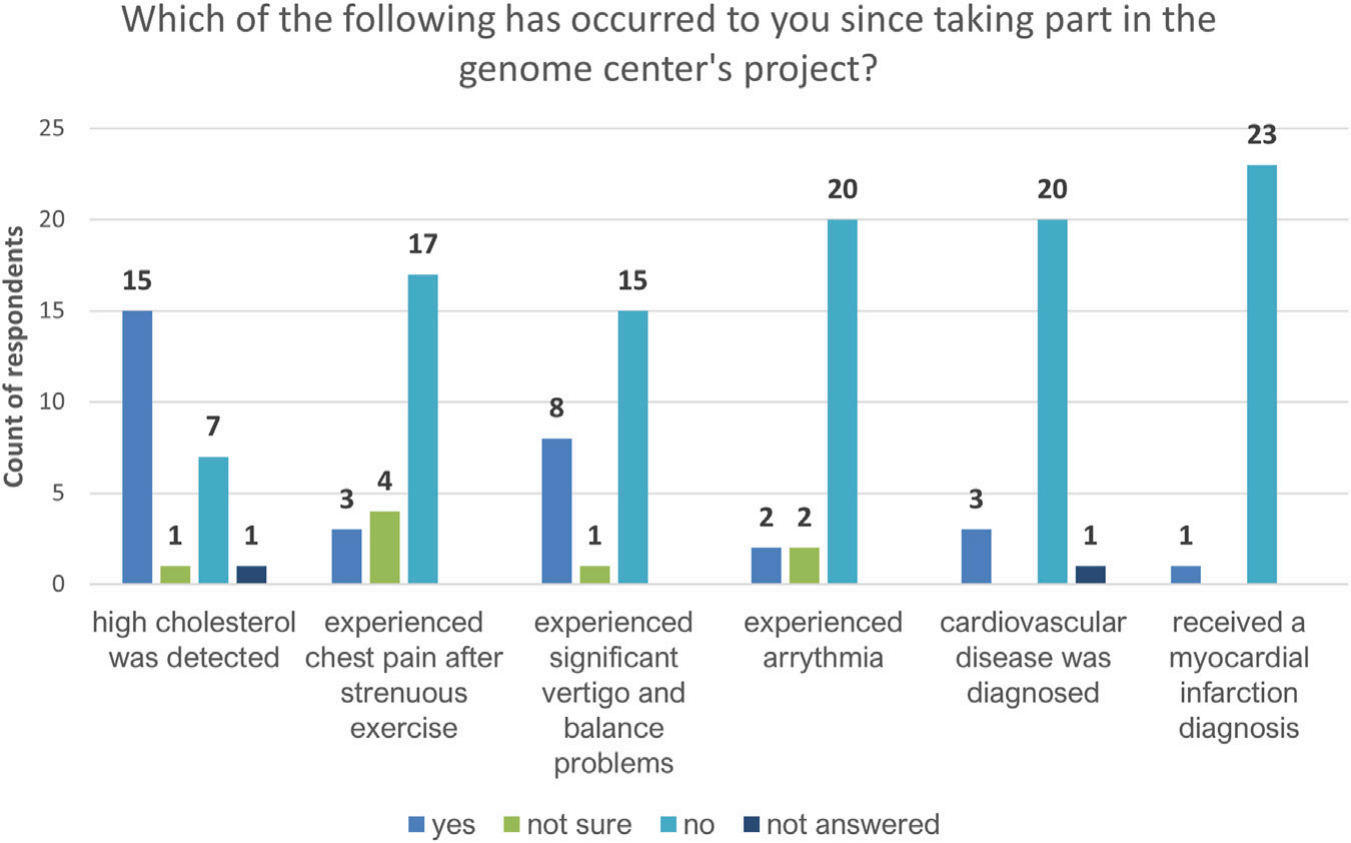
Feedback on health issues potentially related to hypercholesterolemia as reported by recalled participants (*n* = 24).

Regarding potential complications related to FH, the majority of participants indicated only that high cholesterol levels had been detected after their feedback visits (Figure 5). Most respondents reported that no other potential complication (e.g., myocardial infarction, stroke, arrhythmia, chest pain after strenuous exercise, vertigo, balance problems, or cardiovascular disease) had occurred.

**FIGURE 6:**
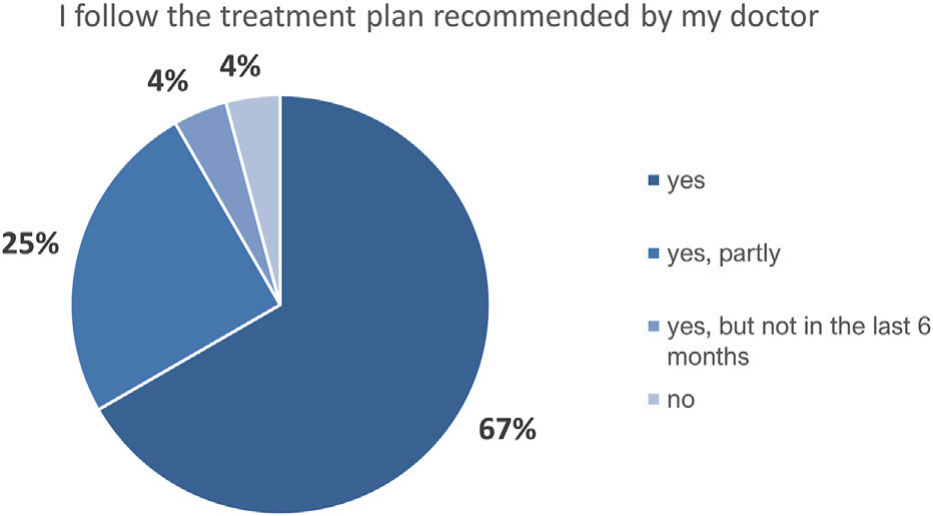
Recalled participants’ self-reported assessment of their adherence to the treatment plan proposed by their doctor (*n* = 24).

**FIGURE 7:**
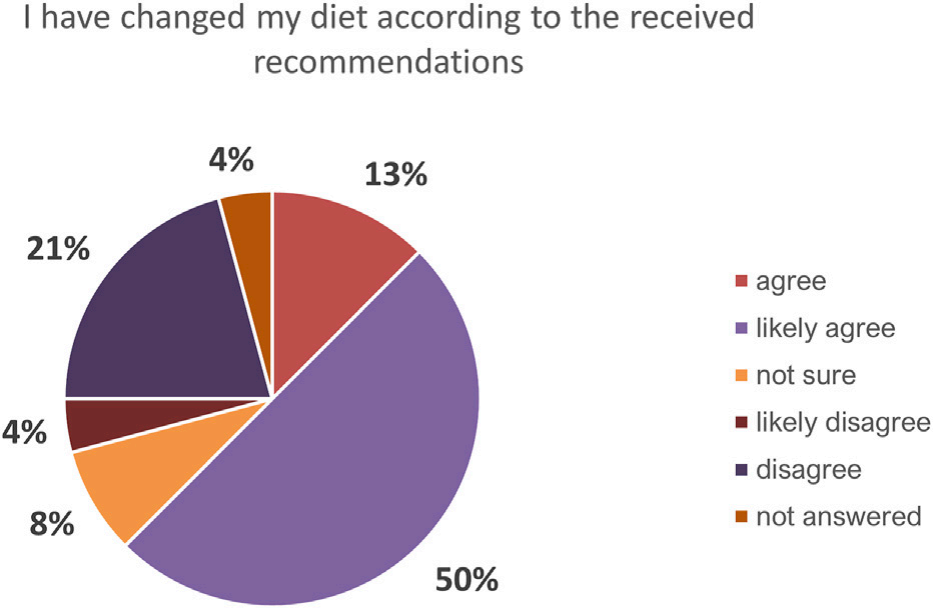
Overview of the feedback to question “How have you changed your habits after receiving information on the genetic variant you carry?” in regard to changes in diet.

Most (92%) participants responded “yes” or “yes, partly” to the question about whether they were following the treatment plans developed with their doctors; 4% responded “yes, but not in the last 6 months” and another 4% reported that they were not following their treatment plans (Figure 6). Nearly two thirds (63%) of respondents reported that they had made at least some changes in their diet after receiving genetic feedback (“likely agree”, 50%; “agree”, 13%). “Disagree,” “likely disagree,” and “unsure” responses to this question made up 21%, 4%, and 8% of the total, respectively. Four percent of the respondents did not answer this question (Figure 7). Responses to questions about changes in participants’ smoking habits and physical activity levels, and with whom they had shared information about their carrier status, are summarized in Supplementary Figures S2–S4.

## Discussion

In this follow-up study, we investigated the impact of the RbG study approach, examining participants’ assessments several years after their genetic findings had been disclosed to them and comparing their characteristics with those of an unrecalled group with similar genetic profiles. Overall, general phenotypic characteristics (i.e., height, weight, body mass index, age, and sex) were comparable in the recalled and non-recalled groups, indicating that the major general confounding factors had little impact on the study results and that the greatest effect on outcomes stemmed from participants’ knowledge of their carrier status.

Similar to the findings reported by Alver et al. (2018), the FH-associated variants identified most frequently in the follow-up cohort were p.Arg3527Gln (rs5742904) in the *APOB* gene and p.Cys329Tyr (rs761954844) in the *LDLR* gene. A possible explanation for the high proportion of *APOB* variants in our cohort could be that rare *LDLR* variants may be technically underrepresented in our genotyped and imputed dataset. To our knowledge, the Alver et al. (2018) study is the only available published overview of FH genetic variants in the Estonian population. No comprehensive overview of the FH-associated genetic variants in Estonian clinical cases has been published.

Significantly larger proportions of the recalled cohort than the control group were diagnosed with pure hypercholesterolemia (ICD-10 code E78.0) and E78* in general, and were prescribed LLTs. Consistent with the conclusions of Alver et al. (2018), these findings indicate that FH remains underdiagnosed and undertreated in Estonia. Despite the recalled participants’ relatively low assessment of follow-up consistency and access to healthcare, these findings also indicate that the feedback visits improved their visibility in the Estonian healthcare system and access to FH treatment compared with the control group, whose disease severity should match that of the recalled cohort. Participants in the recalled cohort also reported very few potential FH complications such as myocardial infarction and stroke, which may be attributable to their earlier receipt of hypercholesterolemia-related diagnoses and statin prescription, when disease progression could still be stalled. However, further investigations are necessary to confirm this hypothesis.

The widespread use of atorvastatin among study participants is not surprising, as it was the best-selling drug in the early 2000s (Kogawa et al., 2019) and remained one of the most popular lipid-lowering medications in more recent years (Pijlman et al., 2010; Casula et al., 2016). In addition, rosuvastatin was among the most popular LLT medications in the study carried out by Casula et al. (2016).

Treatment adherence varied among the small cohort of LLT-using recalled participants; the largest proportion of these participants began using lipid-lowering medications consistently during or shortly after their feedback visits, but another group of nearly the same size had poor overall LLT adherence. The small sample of LLT users may have resulted in greater variance than is likely present in the general population. However, given the generally poor LLT adherence also reported in other studies (Phan et al., 2014; Casula et al., 2016), and several of our participants’ clear improvement in LLT adherence (e.g., #7450009) or consistent LLT use right after the feedback visit (e.g., #7450011), it appears reasonable to speculate that the EstBB intervention had a positive impact on LLT adherence, the full scale of which remains to be captured with larger cohorts.

The third group of LLT users had periods of consistent (≥2 years) adherence prior to the feedback visits, but stopped LLT entirely before 2022. This group contains participants who changed statins at least once; all except one had tried at least three different forms of LLT. Treatment termination was recorded for one participant who died in 2018. Other known reasons for LLT discontinuation were subsequent pregnancies and breastfeeding for a young female participant (despite remarkably high total cholesterol and LDL-C levels in 2016), and myopathy-related side effects that persisted despite several changes in medication over the years reported by another participant. This participant’s pharmacogenomic profile did indicate a higher risk of myopathy as a statin use side effect.

Overall, about 30% of all participants in this study were at greater than normal risk of developing myopathy as a side effect of statin use; about 3% of participants had genotypes corresponding even to a much greater than normal risk. As myopathy is the most predominant statin-related adverse effect and a major reason for poor LLT adherence (Mach et al., 2018), our results suggest the role of pharmacogenomic predisposition and highlight the value of pharmacogenomic profiling for the improvement of LLT adherence.

The survey response rate in this study was 75%, reflecting EstBB contributors’ persisting willingness to cooperate with biobank inquiries, even several years after their feedback visits. This willingness stems partially from participants’ interest in general physical health and awareness of their carrier status, and positive attitudes toward and gratitude for the disclosure of their genetic findings; many participants in the present study indicated that they would have liked to have had knowledge of their genetic findings earlier and most indicated that the choice to receive genetic feedback was good.

When asked to evaluate different aspects of the Estonian healthcare system in the light of their genetic findings, the participants rated their access to medication the highest and indicated that there was room for improvement in the clarity of recommendations, access to healthcare, and follow-up consistency. The participants’ satisfaction with access to medication suggests that the cost or poor supply of medications is not a substantial barrier for patients adhering to their treatment plans in Estonia. Although our study did not estimate the cost-effectiveness of the RbG method, cost-efficiency calculations regarding a variety of genetic prevention services are likely to be performed in the course of the national personalized medicine initiative in Estonia.

Most survey respondents stated that they at least partly followed the treatment plans prescribed by their doctors. Although the importance of LLT in the management of FH is non-negligible (Cuchel et al., 2014), treatment plans for this small cohort did not necessarily involve LLT alone or at all, as dietary changes and various supplements were tried for some time before or together with LLT prescription when patients’ medical histories and clinical characteristics allowed. Accordingly, 63% of the respondents indicated that they had or likely had made changes to their diets according to their doctors’ recommendations. These results, combined with responses indicating a poor understanding of the importance of LLT in general, may explain several participants’ indication that they did follow their treatment plans while having poor LLT adherence.

Granted, there are limitations to our study. The small sample size (and particularly low number of recalled participants that were available to participate in the survey) may lead to a higher variance in results than would be present in a larger cohort.

Additionally, lipid profiles were not available for all non-recalled participants of the control group. As such, we could not confirm the presence of hypercholesterolemia per se in these individuals ourselves but relied on ICD-10 codes (E78* and subsets) and rates of LLT prescription as proxies. Conversely, while the assembled linkage dataset (EHRs, medical prescription registry, etc.) covering a period of approximately 18 years was available for all the participants, an existing deviation from a normal lipid profile, if already diagnosed as a medical problem, would have been detectable. Therefore, by studying this cohort (more than 300 individuals with a confirmed molecular diagnosis of pathogenic or likely pathogenic FH variants) we have reached a turning point to change the situation of FH being underdiagnosed and undertreated in Estonia, and the time to implement nationwide healthcare actions has come.

Based on the current study results, we conclude that future FH screening by genetic testing should be performed more liberally compared to the current clinical practice. Cascade screening would be essential when a new FH case is confirmed. In countries already having large population biobanks and related legislation (including permissive consent forms signed by participants) in place, a large part of the population may receive genome-wide data that could be combined with the main current healthcare strategies. Genetics-first approach with selected additions from usual EHR data builds an empowered rationale for directed pre-screening. We foresee this kind of strategy as a part of personalized medicine or precision prevention. This could enhance the detection of common actionable monogenic disorders (such as FH) much earlier and thereby increase the effectiveness of the prevention activities as well as follow-up of the individuals at risk.

This study demonstrated that the FH RbG study had significant positive impacts on EstBB participants; on the recognition of hereditary dyslipidemia and prescription of clinically indicated LLT for FH-associated variant carriers in the Estonian healthcare system. The importance of recall was further underlined by the participants’ overwhelmingly positive assessment of the utility of the feedback visits. Additionally, the study provided valuable insight into long-term (2004–2022) LLT adherence, suggesting that pharmacogenetic factors (i.e., elevated myopathy risk) play an important role in the generally poor observed adherence. Our results provide a basis for larger-scale RbG studies and add evidence that intervention studies in actionable disorders would be justified in the future.

## Data Availability

The original contributions presented in the study are included in the article/Supplementary Material, further inquiries can be directed to the corresponding author.

## Footnotes

1
https://plot.ly

2
https://langsrud.com/fisher.htm

## Supplementary Material

Supplementary Data Sheet S1 The questionnaire used in recalled participants’ survey in 2021.

Supplementary Table S1 The pharmacogenomic profiles of 27 lipid-lowering treatment using formerly recalled participants.

## Abbreviations

ATC/DDD, Anatomical Therapeutic Chemical/Defined Daily Dose; EHR, electronic health record; EstBB, Estonian Biobank; FH, familial hypercholesterolemia; ICD-10, International Classification of Diseases (10th Revision); LDL-C, low-density lipoprotein cholesterol; LLT, lipid-lowering treatment; RbG, recall-by-genotype; SNP, single nucleotide polymorphism.
