# Supplementary material for: Do Biobank Recall Studies Matter? Long-Term Follow-Up of Research Participants With Familial Hypercholesterolemia
Source: Front Genet. 2022 Jul 19;13:936131. doi: 10.3389/fgene.2022.936131 (PMC9343846; doi:10.3389/fgene.2022.936131)
Supplement: Supplementary file 1 [file DataSheet1.zip › Supplementary_Data1.docx]

*Supplementary Data 1*

**Dear study participant!**

**In 2016-2018, you took part in an earlier scientific study conducted by the Estonian Genome Center of the University of Tartu, „Defining the familial hypercholesterolemia phenotype and subsequent processing in a clinical setting when a genetic finding has been detected in a genome-wide study“. In the course of the project you attended a cardiologist’s consultation and at the end of the study we shared with you the results of our findings.**

**This questionnaire is a continuation of the aforementioned study. By answering this questionnaire, you would provide us with valuable information regarding how you assess now the information you received during the project and whether there have been any notable changes in your health behaviours and habits due to this knowledge. Please answer the following questions by marking the answer you find most suitable. Some questions may already be familiar to you, but do not let this disturb you. We would ask you to mark the answers according to how you are feeling right now.**

**Please recall your earlier decision to receive personalized genetic feedback. Please mark below to what degree you agree with the following statements, by ticking the answer you find most suitable. Please put down an answer for all the statements.**

| It was the right decision | Agree ☐ Slightly agree ☐  Slightly disagree ☐ Disagree ☐ Unsure ☐ |
| --- | --- |
| I regret the choice that was made | Agree ☐ Slightly agree ☐  Slightly disagree ☐ Disagree ☐ Unsure ☐ |
| I would go for the same choice if I had to do it over again | Agree ☐ Slightly agree ☐  Slightly disagree ☐ Disagree ☐ Unsure ☐ |
| The choice did me a lot of harm | Agree ☐ Slightly agree ☐  Slightly disagree ☐ Disagree ☐ Unsure ☐ |
| The decision was a wise one | Agree ☐ Slightly agree ☐  Slightly disagree ☐ Disagree ☐ Unsure ☐ |
| I am able to cope with having this genetic finding  in my family | Agree ☐ Slightly agree ☐  Slightly disagree ☐ Disagree ☐ Unsure ☐ |
| I wish I would have been informed earlier  about the genetic finding and the potential health  risks | Agree ☐ Slightly agree ☐  Slightly disagree ☐ Disagree ☐ Unsure ☐ |
| The information received has somehow  changed my life | Agree ☐ Slightly agree ☐  Slightly disagree ☐ Disagree ☐ Unsure ☐ |
| I now have better access to health care /  specialists | Agree ☐ Slightly agree ☐  Slightly disagree ☐ Disagree ☐ Unsure ☐ |
| I feel that my treatment and/or condition has  improved | Agree ☐ Slightly agree ☐  Slightly disagree ☐ Disagree ☐ Unsure ☐ |

**Have you shared information on your genetic finding with anybody?**

Spouse/partner Yes ☐ Not yet, but planning to ☐ No ☐ Not possible ☐

Children Yes ☐ Not yet, but planning to ☐ No ☐ Not possible ☐

Siblings Yes ☐ Not yet, but planning to ☐ No ☐ Not possible ☐

Parents Yes ☐ Not yet, but planning to ☐ No ☐ Not possible ☐

Physician Yes ☐ Not yet, but planning to ☐ No ☐

Other _______________ Yes ☐ Not yet, but planning to ☐ No ☐

**If you shared information regarding your genetic finding, what was the response?**

____________________________________________________________________________________________________

**If you are not planning to share information regarding the genetic finding with anyone, what is the main reason for doing so?**

____________________________________________________________________________________________________

**Which of the following has occurred to you since taking part of the genome center’s project?**

high cholesterol was detected Yes ☐ No ☐ Unsure ☐

cardiovascular disease was diagnosed Yes ☐ No ☐ Unsure ☐

experienced significant vertigo and balance problems Yes ☐ No ☐ Unsure ☐

experienced arrythmia Yes ☐ No ☐ Unsure ☐

experienced chest pain after strenuous exercise Yes ☐ No ☐ Unsure ☐

received a myocardial infarction diagnosis Yes ☐ No ☐ Unsure ☐

received a stroke diagnosis Yes ☐ No ☐ Unsure ☐

**Which of the following activities have you done since participating in genome center’s familial hypercholesterolemia project? Please respond to each of the statements by marking the most appropriate answer.**

I have recommended my family members to get a doctor consultation

Yes ☐ Not yet, but planning to ☐ No ☐

I am being monitored by my family physician or a specialist doctor regarding familial hypercholesterolemia.

Yes, I have seen a doctor regarding this matter within the past year ☐

Yes, but it has been more than a year yet less than two years since the last visit ☐

No, I have not seen a doctor regarding this matter within the past two years ☐

No, I have not seen a doctor regarding this matter since the participating in the project ☐

No, I have not seen any doctors since the project ☐

I follow the treatment-plan recommended by my doctor

Yes ☐

Yes, partially ☐

Yes, but not within the past 6 months ☐

No, I stopped more than 6 months ago ☐

No ☐

**How would you rate your experience with the healthcare received in regard to your genetic finding?**

Access to care Very good ☐ Good ☐ Satisfactory ☐ Unsatisfactory ☐ Unsure ☐

Consistency of follow-up Very good ☐ Good ☐ Satisfactory ☐ Unsatisfactory ☐ Unsure ☐

Clarity of recommendations Very good ☐ Good ☐ Satisfactory ☐ Unsatisfactory ☐ Unsure ☐

Access to medications Very good ☐ Good ☐ Satisfactory ☐ Unsatisfactory ☐ Unsure ☐

What did you appreciate the most?

________________________________________________________________________________________________

________________________________________________________________________________________________

________________________________________________________________________________________________

What has been the greatest shortcoming? ________________________________________________________________________________________________

________________________________________________________________________________________________

________________________________________________________________________________________________

**How have you changed your habits after receiving information on the genetic variant you carry?**

Smoking Increased ☐

Decreased ☐

Same ☐

Unsure ☐

Never smoked ☐

Quit smoking ☐

Physical activity Increased ☐

Decreased ☐

Same ☐

Unsure ☐

I have changed my diet according to the received recommendations

Agree ☐

Slightly agree ☐

Slightly disagree ☐

Disagree ☐

Unsure ☐

Other _____________________________________________________________________________________

**Where have you sought additional information from? Please mark all that apply.**

Asked my physician ☐ From the Internet ☐ Other _________________________

I have not sought additional information ☐

**Do you have any recommendations on what the genome center could have done differently during the familial hypercholesterolemia project?**

__________________________________________________________________________________________
__________________________________________________________________________________________

**Do you have any recommendations or suggestions on what could be done differently in the current health care system when a person with a familial hypercholesterolemia genetic variant turns to a doctor?**

__________________________________________________________________________________________

_______________________________________________________________________________________

**Thank You!**
