# Supplementary material for: Do Biobank Recall Studies Matter? Long-Term Follow-Up of Research Participants With Familial Hypercholesterolemia
Source: Front Genet. 2022 Jul 19;13:936131. doi: 10.3389/fgene.2022.936131 (PMC9343846; doi:10.3389/fgene.2022.936131)
Supplement: Supplementary file 1 [file DataSheet1.zip › Supplementary_Figures.docx]

*Supplementary Figures*


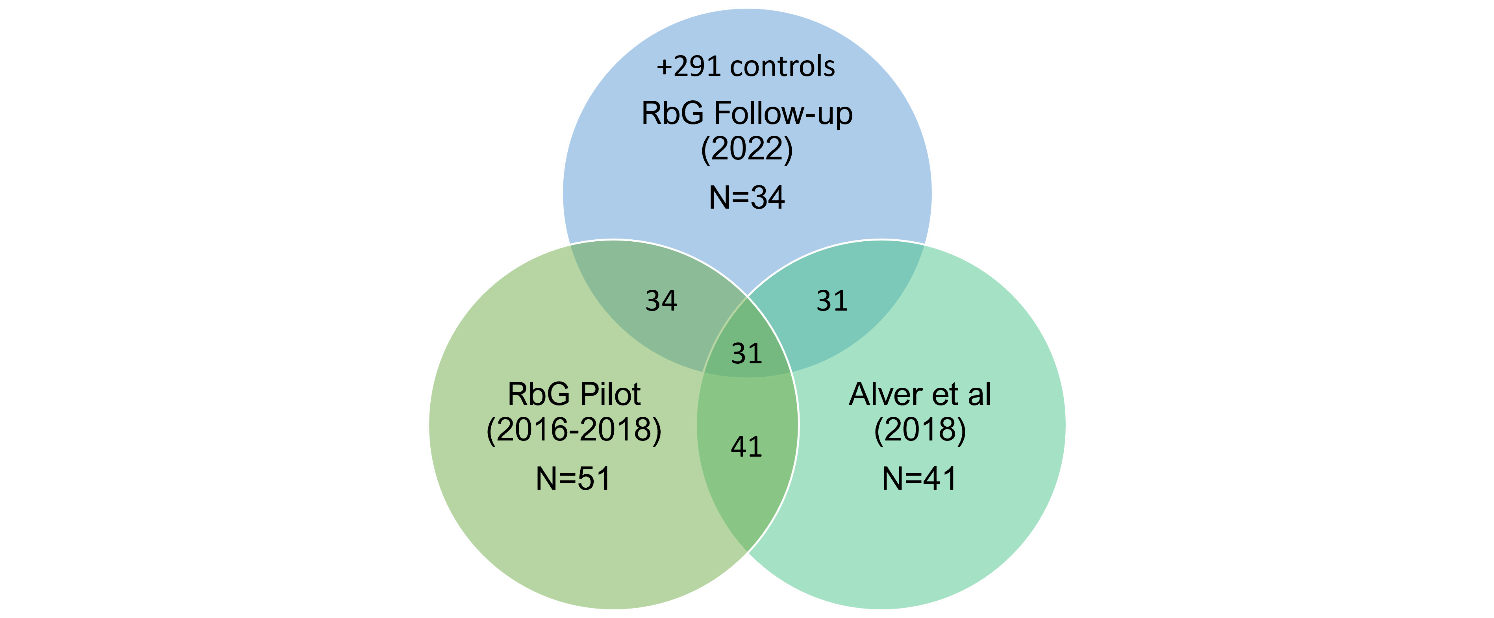


**Supplementary Figure S1**. Repartition of recalled participants across the RbG pilot study, follow-up study and publicized results from Alver et al (2018). Only the recalled participants from the RbG pilot study who had signed up as biobank participants by fall 2021, were eligible to participate in the follow-up study.


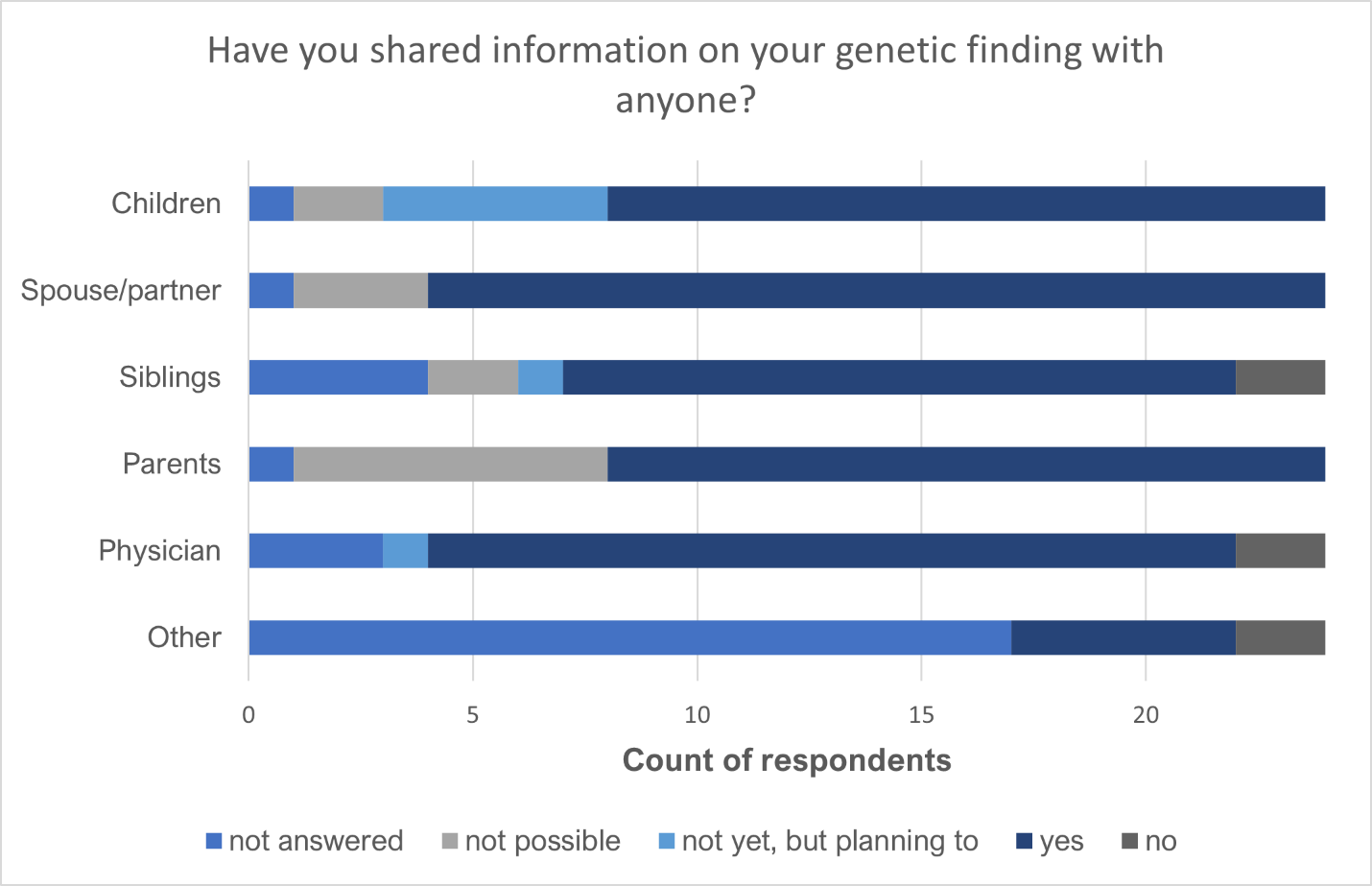


**Supplementary Figure S2**. Overview of participants’ feedback about whether they have shared knowledge about their genetic finding and with whom (n=24).


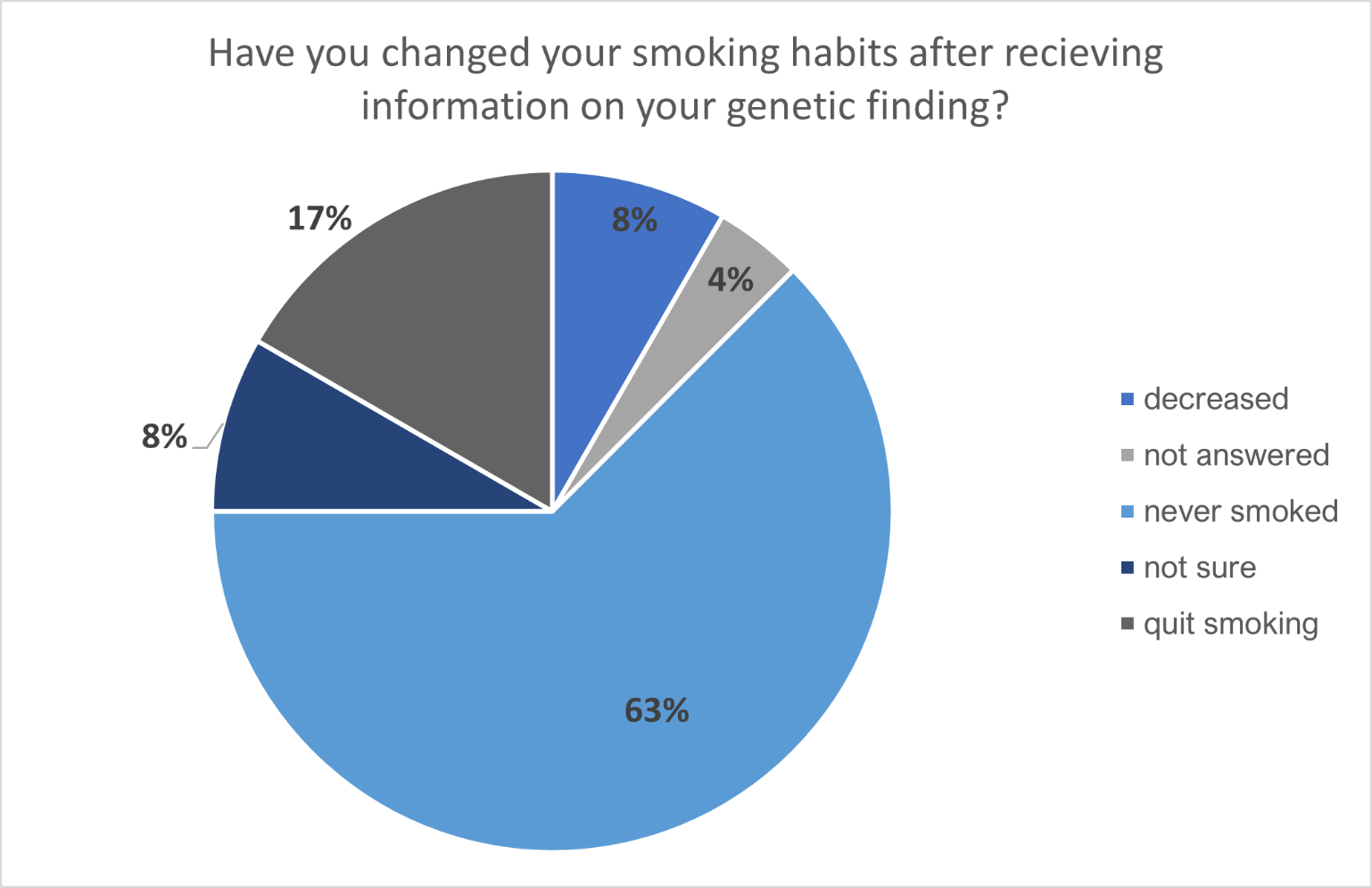


**Supplementary Figure S3.** Overview of participants’ feedback on how their smoking habits have changed after receiving genetic feedback (n=24).


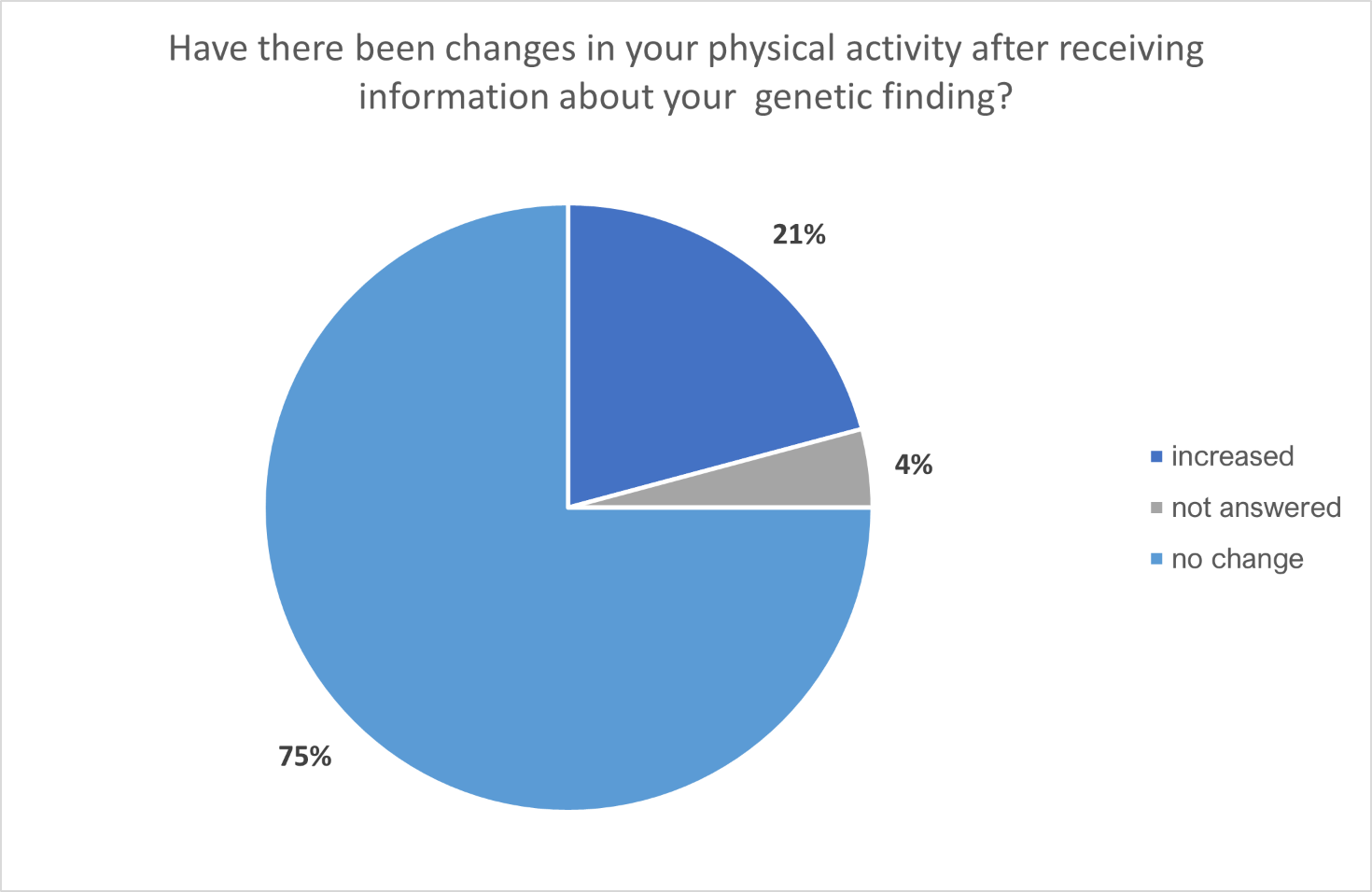


**Supplementary Figure S4.** Overview of participants’ assessment on how their physical activity level has changed after receiving genetic feedback (n=24).
